# Visualizing the Bohr effect in hemoglobin: neutron structure of equine cyanomethemoglobin in the R state and comparison with human deoxyhemoglobin in the T state

**DOI:** 10.1107/S2059798316009049

**Published:** 2016-06-28

**Authors:** Steven Dajnowicz, Sean Seaver, B. Leif Hanson, S. Zoë Fisher, Paul Langan, Andrey Y. Kovalevsky, Timothy C. Mueser

**Affiliations:** a Department of Chemistry and Biochemistry, University of Toledo, Toledo, Ohio, USA; b Scientific Activities Division, Science Directorate, European Spallation Source, PO Box 176, 221 00 Lund, Sweden; c Biology and Soft Matter Division, Oak Ridge National Laboratory, Oak Ridge, Tennessee, USA

**Keywords:** neutron crystallography, cyanomethemoglobin, alkaline Bohr effect, H/D exchange

## Abstract

Neutron crystallography provides direct visual evidence of the atomic positions of deuterium-exchanged H atoms, enabling the accurate determination of the protonation/deuteration state of hydrated biomolecules. Comparison of two neutron structures of hemoglobins, human deoxyhemoglobin (T state) and equine cyanomethemoglobin (R state), offers a direct observation of histidine residues that are likely to contribute to the Bohr effect. Previous studies have shown that the T-state N-terminal and C-terminal salt bridges appear to have a partial instead of a primary overall contribution. Four conserved histidine residues [αHis72(EF1), αHis103(G10), αHis89(FG1), αHis112(G19) and βHis97(FG4)] can become protonated/deuterated from the R to the T state, while two histidine residues [αHis20(B1) and βHis117(G19)] can lose a proton/deuteron. αHis103(G10), located in the α_1_:β_1_ dimer interface, appears to be a Bohr group that undergoes structural changes: in the R state it is singly protonated/deuterated and hydrogen-bonded through a water network to βAsn108(G10) and in the T state it is doubly protonated/deuterated with the network uncoupled. The very long-term H/D exchange of the amide protons identifies regions that are accessible to exchange as well as regions that are impermeable to exchange. The liganded relaxed state (R state) has comparable levels of exchange (17.1% non-exchanged) compared with the deoxy tense state (T state; 11.8% non-exchanged). Interestingly, the regions of non-exchanged protons shift from the tetramer interfaces in the T-state interface (α_1_:β_2_ and α_2_:β_1_) to the cores of the individual monomers and to the dimer interfaces (α_1_:β_1_ and α_2_:β_2_) in the R state. The comparison of regions of stability in the two states allows a visualization of the conservation of fold energy necessary for ligand binding and release.

## Abstract 2

The determination of the positions of H/D atoms in equine cyanomethemoglobin by neutron diffraction is described.

## 1. Introduction   

Hemoglobin (Hb) is one of the best-characterized macromolecules and is regarded as an ideal model for studies of protein evolution, cooperativity and allostery. In vertebrates, two heterodimers (α_1_β_1_:α_2_β_2_) comprise the oxygen-transporting functional tetramer contained at high concentration (∼5 m*M* tetramer, normal mean corpuscular hemoglobin per mean corpuscular volume) inside the red blood cells (RBCs). This containment is crucial since the tightly coupled deoxy tetramer (*K*_d_ ≃ 0.01 n*M*) would dissociate into liganded relaxed (R-state) dimers (*K*_d_ ≃ 1 µ*M*) at lower concentrations (Atha & Riggs, 1976 ▸). Binding of oxygen in the lung, driven by the increased partial pressure of oxygen, the tetramer transitions into the fully liganded R state. As RBCs move away from the lungs, the partial pressure of oxygen declines, leading to ligand dissociation, promoted by the stabilizing effect of the deoxy tense (T-state) tetramer. In the tissue capillaries, the RBCs also encounter an increase in the concentration of dissolved CO_2_ and a corresponding decrease in pH, where hemoglobin assists in the transport of CO_2_ and excess protons. Several heterotropic allosteric hemoglobin modulators, including H^+^, CO_2_ and 2,3-bisphosphoglycerate (2,3-BPG), regulate the reversible binding of O_2_. The release and uptake of protons by hemoglobin is known as the ‘Bohr effect’ and ionizable residues influenced by this effect are known as ‘Bohr groups’. Perutz and coworkers proposed that salt bridges present in the T state primarily influence the Bohr effect. The salt bridges involve the α N-terminus, Val1(NA1), bridged to the α C-terminus, Arg141(HC3), and the β C-terminus, His146(HC3), bridged to both αLys40(C6) and βAsp94(FG1) (globin fold positions are denoted in parentheses; Perutz *et al.*, 1969 ▸, 1980 ▸; Perutz, 1970 ▸). Multiple studies have implicated additional residues that contribute to the Bohr effect (Berenbrink, 2006 ▸; Lukin & Ho, 2004 ▸). From a structural point of view, the transition from the R state to the T state alters the local environments of potential Bohr groups. As expected, small changes in relative positions shift the p*K*_a_ values of ionizable residues, altering the proton affinity of a particular Bohr group (Thurlkill *et al.*, 2006 ▸). Despite the numerous theoretical and experimental studies on hemoglobin, the specific changes in the protonation state of potential Bohr groups in the R and T states remains uncertain, as the techniques that have previously been utilized do not provide direct observation of the protonation state.

The cooperative binding behavior of O_2_ to Hb has been investigated both kinetically and thermodynamically (Parkhurst, 1979 ▸; Ackers, 1980 ▸). In most models, Hb is proposed to exist in two states: a high O_2_ affinity R state and a low O_2_ affinity T state. The two states are revealed in the biphasic on-rates of O_2_ (33 and 7 µ*M* ^−1^ s^−1^ for the R and T states, respectively) and in the sigmoidal shape of the oxygen-dissociation curves (Parkhurst, 1979 ▸; Imai *et al.*, 1970 ▸). Detailed analysis of oxygen-dissociation curves has yielded a number of mathematical models for the R-to-T transition. These include the Hill equation, where all monomers are treated equally, the Adair equation, where individual equilibrium constants are introduced, and the symmetry model (extended Adair equation), where the R and T states are included in the binding steps (Parkhurst, 1979 ▸). The introduction of induced-fit and free-energy penalties for subunit association provided the best overall understanding of the thermodynamics of oxygen delivery supported by the view of hemoglobin provided by structural analysis (Ackers, 1980 ▸; Perutz *et al.*, 1980 ▸; Koshland *et al.*, 1966 ▸). In Acker’s free-energy penalty model, the presence of ligand affects the stability of dimer interfaces (α_2_:β_1_ and α_1_:β_2_), with the greatest strain introduced when ligands bind on opposing dimers (Ackers, 1980 ▸). The first ligand-binding step is restricted owing to the tetramer stability of the T state, while the last ligand has high affinity owing to the transfer of stability to the dimer. The free-energy difference between the two states is ∼6.3 kcal mol^−1^, where the T-state tetramer is more thermodynamically stable. Structurally, the oxygenation of Hb disrupts major dimer–dimer interactions and stabilization is shifted towards the individual monomers.

**Figure 1:**
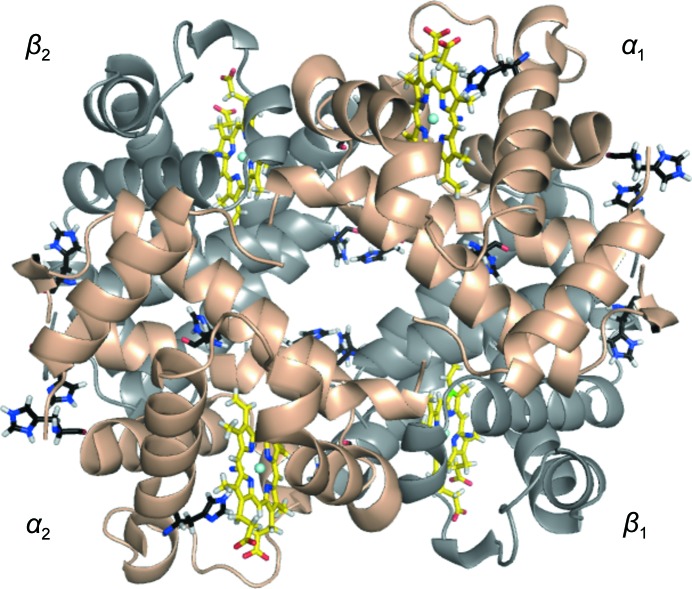
Overall structure of EqCNmetHb: a ribbons model of EqCNmetHb with α subunits in wheat, β subunits in gray, heme groups in yellow and His residues in black.

The Bohr effect is the linkage of decreasing oxygen affinity to increasing CO_2_ concentration, mediated by the formation of carbaminohemoglobin and the acidity of the dissolved CO_2_, where proton binding influences the release of oxygen. Approximately two CO_2_ molecules and two H^+^ ions are added to the Hb tetramer during the R-to-T transition (O’Donnell *et al.*, 1979 ▸; Perutz *et al.*, 1980 ▸). In order to identify a potential Bohr group, the protonation state of histidine (His) needs to be established in both the T and the R state (Fig. 1 ▸). These His residues are titratable at neutral pH. Comprehensive studies using NMR, H/D exchange and site-directed mutagenesis of recombinant Hb have determined p*K*_a_ differences for 24 of the 38 possible Bohr-group His residues (Sun *et al.*, 1997 ▸; Fang *et al.*, 1999 ▸; Ohe & Kajita, 1980 ▸). Recent p*K*_a_ calculations based on high-resolution crystallographic models have provided a detailed prediction of the protonation states of the Bohr residues (Park *et al.*, 2006 ▸).

This study directly visualizes the positions of exchangeable H atoms in equine cyanomethemoglobin (EqCNmetHb; Fig. 1 ▸) using neutron crystallography and deuterium exchange. EqCNmetHb is a stable model for the liganded hemoglobin R state crystallized at physiological pH. Equine hemoglobin has 88% sequence identity to human hemoglobin and is considered to be isomorphous with human oxyhemoglobin, with most histidine positions conserved (Ladner *et al.*, 1977 ▸). A 2.0 Å resolution neutron data set was collected at the Protein Crystallography Station using the pulsed spallation neutron source of the Los Alamos Neutron Science Center (Langan *et al.*, 2004 ▸, 2008 ▸). Comparison of this R-state model with the previously reported neutron crystal structure of human deoxyhemoglobin (PDB entry 3kmf; HuDeoxyHb) provides a unique opportunity to compare and contrast the extent of protonation/deuteration (H^+^/D^+^) of the R and T states (Kovalevsky, Chatake *et al.*, 2010 ▸). While obviously more appropriate, crystals of human R-state Hb are not amenable to neutron diffraction analysis owing to smaller crystal size and an excessively long unit-cell dimension (space group *P*4_1_2_1_2, unit-cell parameters *a* = 53.7, *c* = 193.5 Å). The use of equine Hb as the model R-state hemoglobin (space group *C*2, unit-cell parameters *a* = 108.8, *b* = 63.0, *c* = 54.6Å, β = 110.7°) has long been established, and for long-term ligand stability the use of ferricyanide was essential (Perutz, 1968 ▸).

Variations in histidine H^+^/D^+^ states are coupled to changes in the local environment involving changes in solvent accessibility, changes in hydrogen-bond networks and disruption of proton shuttles. Comparison of the two neutron structures provides proper orientation and discerns changes in the histidine rings while also providing a qualitative assessment of the presence or absence of H^+^/D^+^ at the δ and ∊ positions. These studies indicate that six histidine residues show significant changes in their H^+^/D^+^ states, with some gaining protons, supporting the Bohr effect, while others lose protons, in opposition. The backbone amides of the taut state (deoxy T state) exhibit a comparable level of non-exchangable H atoms to that seen in the relaxed state (liganded R state). Interestingly, the regions of non-exchangeability change location, providing a direct observation of the preservation of fold energy during ligand binding. These differences in backbone exchange indicate an increase in flexibility of the R-state α_1_:β_2_ interface, with a corresponding decrease in the flexibility of the α_1_:β_1_ interface and the heme pocket correlated to ligand binding.

## 2. Methods   

### 2.1. Crystallization and data collection and reduction   

EqCNmetHb crystallization was carried out using the batch method at pH 7.2 in a nine-well depression plate (Hampton Research) as described previously (Mueser *et al.*, 2000 ▸; Kovalevsky, Fisher *et al.*, 2010 ▸). This method produced several large crystals; the crystal used in this study had a volume of ∼10 mm^3^ (Kovalevsky, Fisher *et al.*, 2010 ▸). The EqCNmetHb crystal underwent H/D vapor exchange in a quartz capillary for one month before data collection (Kovalevsky, Fisher *et al.*, 2010 ▸). A neutron time-of-flight Laue diffraction experiment was carried out at the Protein Crystallography Station (PCS) located at Los Alamos Neutron Scattering Center (LANSCE). The data-collection and neutron data-reduction details have been described previously (Kovalevsky, Fisher *et al.*, 2010 ▸). After the neutron data set had been collected, the crystal was subjected to X-ray data collection and the X-ray data were initially integrated and scaled using d*TREK during data collection (Pflugrath, 1999 ▸) as described previously (Kovalevsky, Fisher *et al.*, 2010 ▸). During joint refinement, it became apparent that the previously reported X-ray data were not optimally processed. Therefore, reintegration and rescaling of the X-ray diffraction data was performed using *iMosflm* and *SCALA* incorporated into the *CCP*4 program suite (Evans, 2006 ▸; Winn* et al.*, 2011 ▸; Leslie, 2006 ▸). The reprocessed data have a slightly higher overall *R*_merge_ than the previously reported data. However, the crystal mosaicity was significantly lower (0.42 *versus* 1.36°), with a higher signal-to-noise ratio (12.9 *versus* 10.9 overall; 3.9 *versus* 2.1 in the highest resolution shell). The reprocessed data also produced noticeably better quality maps in both X-ray and joint refinement.

### 2.2. Structure determination and refinement   

The room-temperature (RT) X-ray structure was refined using *REFMAC*5 from the *CCP*4 program suite (Murshudov *et al.*, 2011 ▸); the initial starting model for rigid-body refinement was PDB entry 1g0b (Mueser *et al.*, 2000 ▸; Kovalevsky, Fisher *et al.*, 2010 ▸) and the statistics are highlighted in Table 1 ▸. This RT X-ray structure was later used as the starting model for the joint refinement process. The joint X-ray/neutron refinement was executed in *nCNS*, starting with rigid-body refinement followed by several cycles of positional, atomic displacement parameter (*i.e.* *B*-factor) and D-occupancy refinement (Adams *et al.*, 2009 ▸). Between each refinement cycle, D atoms and D_2_O molecule positions and orientations were manually adjusted using *Coot* (Emsley & Cowtan, 2004 ▸). In *Coot*, the orientation and the H^+^/D^+^ state of each ionizable residue were inspected with nuclear |*F*_o_| − |*F*_c_| and 2|*F*_o_| − |*F*_c_| Fourier X-ray density maps with σ_A_-type weights. In order to provide evidence of the protonation/deuteration state of specific side chains (His, Lys, Glu, Asp, Tyr, Thr and Ser), D atoms were deleted from the model, the structure was refined and the omitted sections were inspected again by calculating |*F*_o_| − |*F*_c_| and 2|*F*_o_| − |*F*_c_| maps. Positive density peaks at the location of the omitted atoms permitted direct determination of the deuteration states of specific side chains. For refinement purposes, disordered side chains (lack of both electron and neutron density) were assigned based upon their p*K*_a_ at pH 7.4. D_2_O molecules were assigned and built into the positive density peaks during X-ray refinement and orientated based upon hydrogen bonding and the nuclear |*F*_o_| − |*F*_c_| and 2|*F*_o_| − |*F*_c_| density maps. The figures and solvent-accessibility percentages were generated using *PyMOL*. The solvent-accessibility percentage was estimated using the ‘get_area’ option in *PyMOL* (dot density = 4, dot_solvent = on). Side-chain exposure was calculated by differencing values with (histidine) and without (alanine) the imidazole ring.

### 2.3. Hydrogen/deuterium-exchange (HDX) analysis   

The scattering lengths of D and H are 6.67 × 10^−15^ and −3.74 × 10^−15^ m, respectively, which results in the scattering length of H equalling −0.56 of the D scattering length. During the individual occupancy refinement in *nCNS* the minimum value associated with the occupancy of an atom is set to −0.56 (Adams *et al.*, 2009 ▸). At this point in the refinement the atomic displacement parameters are constrained. Because of the chemical nature of a peptide bond, the backbone amide proton has the ability to exchange with D_2_O. In the case of an amide proton that is stabilized by a strong hydrogen-bonding network, the proton will be shielded from exchange with D_2_O. For the individual occupancy refinement the occupancy of the backbone amide protons can refine to between −0.56 and 1.0, with −0.56 if the amide proton is H and 1.0 if it is D. The refined backbone amide proton occupancies were analyzed for EqCNmetHb and HuDeoxyHb, with occupancies ranging from −0.56 to 0 assigned as a non-exchanged proton, occupancies of 0–0.6 assigned as partially exchanged H and occupancies of 0.6–1.0 assigned as fully exchanged D. These results from the individual occupancy refinement allow the areas of stability and dynamics in the two Hb neutron structure compared here to be visualized.

## 3. Results and discussion   

### 3.1. Neutron structure determination of EqCNMetHb   

EqCNmetHb crystals are inherently more stable and easier to handle than equine carbonmonoxy-Hb crystals (PDB entry 1g0b), allowing large crystals (>5 mm^3^) to be obtained (Mueser *et al.*, 2000 ▸; Kovalevsky, Fisher *et al.*, 2010 ▸). The neutron time-of-flight (TOF) data-collection and refinement statistics are outlined in Table 1 ▸. The EqCNmetHb neutron structure was solved to 2.0 Å resolution using joint X-ray/neutron refinement, a resolution sufficient to accurately locate D atoms in macromolecules (Adams *et al.*, 2009 ▸; Blakeley *et al.*, 2008 ▸). The resultant real-space final 2|*F*_o_| − |*F*_c_| nuclear density maps allowed the assignment of most labile H atoms, with the exceptions being a few highly disordered, solvent-exposed side chains that are also absent in the electron-density maps. While the diffraction resolution is not high enough to accurately determine partial occupancies, they can establish the presence or absence of D (positive nuclear density peaks) or the presence of a non-exchanged H (negative nuclear density peaks). All Asp and Glu residues were interpreted as deprotonated. The majority of hydroxyl-containing side chains (Thr, Ser and Tyr) showed positive peaks for D atoms and were interpreted as exchanged with D_2_O. The exchange rate of tyrosine hydroxyl groups is relatively rapid and decreases as a function of strong hydrogen-bond networks associated with the side chain (Takeda *et al.*, 2009 ▸). For a few residues, αTyr24(B5), αThr38(C3), αSer81(F2), αSer131(H14), βSer44(CD3) and βSer70(E14), the hydroxyls did not show positive deuteration peaks, suggesting the absence of exchange. However, these groups are located on the surface and lack any strong hydrogen-bond networks; therefore, exchange with D is likely but perhaps may not be visible owing to a high freedom of motion.

**Figure 2:**
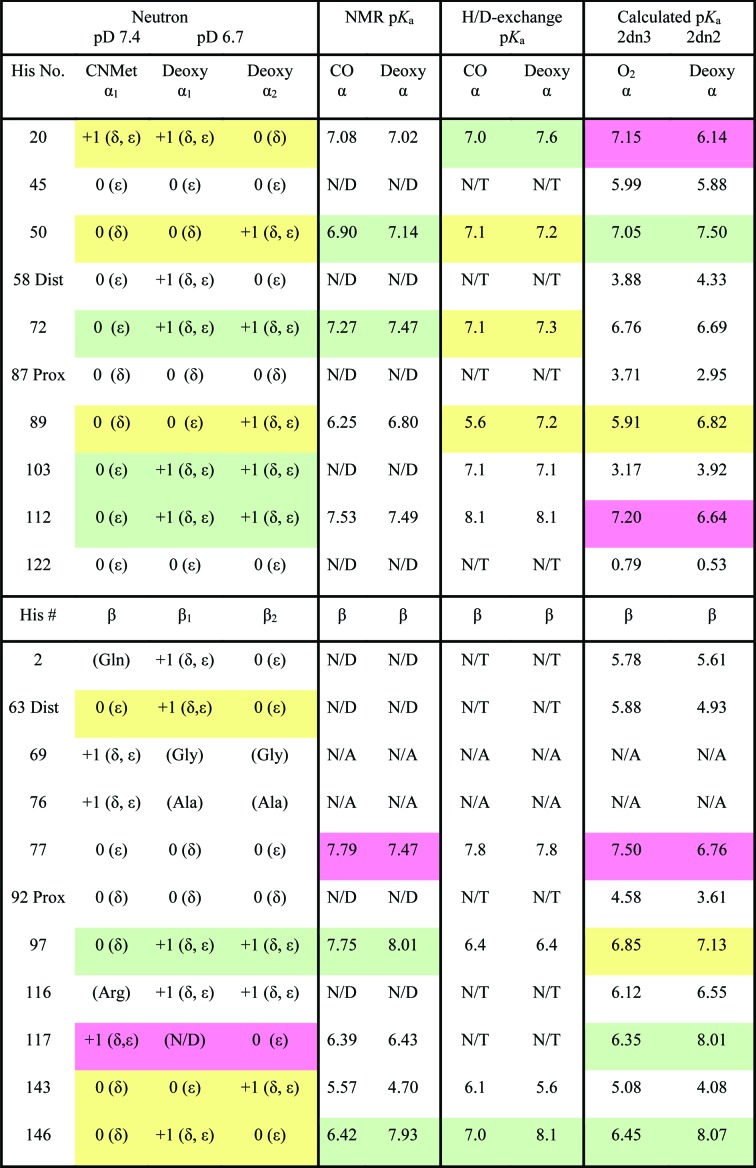
Protonation states of histidine residues in deoxyHb *versus* CNmetHb with p*K*_a_ values determined by previous NMR and H/D-exchange studies, and by molecular mechanics calculations based on high-resolution crystal structures. The protonation states of His residues are indicated by their charge: ‘0’ refers to a neutral His side chain (imidazole ring), while +1 indicates that the His side chain is protonated (formal positive charge). The position of the D atom is indicated by δ and ∊ for His N^δ^ and N^∊^, respectively. Values of p*K*_a_ are calculated from the ^1^H NMR spectroscopic measurements published by Sun *et al.* (1997 ▸) and Fang *et al.* (1999 ▸). N/D, not determined; N/A, not applicable. H/D-exchange studies published by Ohe & Kajita (1980 ▸) calculated the p*K*_a_ values for the individual His residues for deoxy and CO human hemoglobin. N/T, not titratable. The p*K*_a_ values calculated by molecular mechanics for oxy Hb (PDB 2dn3) and deoxy Hb (PDB 2dn2) were published by Zheng *et al.* (2013 ▸). The color-coding indicates support of the Bohr effect (green), marginal support (yellow) and counter-support (red).

The equine hemoglobin tetramer has 38 His residues: 20 in the α chains and 18 in the β chains. The *C*2 symmetry of the EqCNmetHb crystals aligns the molecular dyad with a crystallographic axis, reducing the asymmetric unit to an αβ dimer and the number of His residues to analyze to ten for the α chain [residues 20(B1), 45(CD3), 50(CD8), 58(E7 distal), 72(EF1), 87(F8 proximal), 89(FG1), 103(G10), 112(G19) and 122(H5)] and nine for the β chain [residues 63(E7 distal), 69(E13), 76(E20), 77(EF1), 92(F8 proximal), 97(FG4 switch), 117(G19), 143(H21) and 146(HC3)]. The His positions in the equine (Eq) α chains are identical to those in HuDeoxyHb (Hu), whereas those in the β chains differ at four locations: Eq Gln2/Hu His2(NA2), Eq His69/Hu Gly69(E13), Eq His76/Hu Ala76(E20) and Eq Arg116/Hu His116(G18) (Fig. 2 ▸). Histidines have a slightly acidic p*K*_a_ (∼6.8) on average with environmental variability, with buried side chains becoming more acidic. EqCNmetHb crystals were grown at pH 7.2 (pD 7.6), where a solvent-exposed His is expected to have N^∊2^ fully occupied with H^+^/D^+^ and N^δ1^ partially occupied with H^+^/D^+^ (∼2.5:1 His^0^:His^+^). In the EqCNmetHb neutron structure the His residues were initially doubly occupied and the occupancy values were refined (ranging from 0 to 1) to estimate His^0^:His^+^ ratios with the final model containing the predominant form. Neutral histidine (His^0^) can exist in two tautomeric states with H^+^/D^+^ either at the N^δ1^ or N^∊2^ position, with N^∊2^ being the more likely (Fig. 2 ▸). Two conserved His residues [αHis20(B1) and βHis117(G19)] were determined to be doubly protonated (His^+^), with the occupancies of both D atoms refining to a value of 1. The H^+^/D^+^ occupancies of these two residues are clearly influenced by neighboring conserved acidic groups: αHis20(B1) is hydrogen-bonded to αGlu23(B4) and βHis117(G19) is hydrogen-bonded to βGlu26(B8). Interestingly, two His residues that are unique to equine Hb, βHis69(E13) and βHis76(E20) (Gly and Ala in human Hb, respectively), are the only others that appear to be doubly protonated in the R state and are also influenced by neighboring groups: βHis69(E13) is hydrogen-bonded to βGlu20(B2) (Val in human) and to βGlu73(E16) (Asp in human), and βHis76(E20) is buried in a crystal contact. Four His residues appear to be disordered [αHis50(CD8), αHis89(FG1), βHis143(H21) and βHis146(HC3)] and were assigned to be neutral (His^0^) with N^∊2^ occupied by H^+^/D^+^. All other His residues in the EqCNmetHb neutron structure were determined to be singly H^+^/D^+^-occupied (His^0^). The proximal His residues, αHis87(F8) and βHis92(F8), have N^∊2^ coordinated to the Fe ion and N^δ1^ exchanged with D. Of the remaining nine His residues, all have N^∊2^ occupied with H^+^/D^+^ as expected, with the exception of βHis97(FG4 switch), which has N^δ1^ occupied with H^+^/D^+^ (Fig. 2 ▸).

### 3.2. Comparison of R and T states and visualization of the Bohr effect   

The availability of two neutron structures (EqCNmetHb and HuDeoxyHb) allows direct visualization of the changes in the hydrogen-bonding networks associated with the potential Bohr groups. Essential experimental conditions impose caveats on this analysis as listed. (i) The use of equine Hb instead of human Hb as the R-state model was required to obtain crystals that were suitable for neutron diffraction. (ii) The duration of the studies, several months for deuterium exchange and data collection, necessitated the use of the stable ferric cyanomet derivative instead of a ferrous heme with CO or O_2_. (iii) Crystallization of the HuDeoxyHb crystal was completed at pH 6.3 (pD 6.7), which potentially exaggerates Bohr-group titration, while the R-state pH was completed at closer to the physiological conditions (pH 7.2, pD 7.6).

The pH/pD difference between crystal forms can account for changes in the protonation states of several His residues and is noted when relevant. Comparisons of His-residue protonation in the R-state and T-state models are tabulated in Fig. 2 ▸ along with the published p*K*_a_ values estimated by NMR, H/D exchange and molecular mechanics calculations (Sun *et al.*, 1997 ▸; Fang *et al.*, 1999 ▸; Ohe & Kajita, 1980 ▸; Zheng *et al.*, 2013 ▸). The p*K*_a_ values and shifts determined by NMR and H/D exchange have discrepancies, and predictions of protonation do not necessarily correlate with what is seen in the neutron structures. For example, the p*K*_a_ value determined by NMR and H/D exchange suggests that no histidines in the R state would be doubly protonated. However, two conserved His residues [αHis20(B1) and βHis117(G19)] clearly show protonation at both the N^δ1^ and N^∊2^ positions. Also, the lower pH of the T-state crystal should have the primary Bohr groups, βHis146(HC3), both doubly protonated. Instead, the two crystallographically distinct C-termini, un­affected by crystal contacts, show variation: one is doubly protonated, while the other is singly protonated, suggesting a more modest involvement in the overall Bohr effect. The values of p*K*_a_ are not accurately determinable from the deuterium occupancies of titratable groups. Instead, visualization of the changes in local environment that affect hydrogen-bonding networks correlates to the changes in p*K*_a_ values, especially in residues with limited solvent accessibility (Supplementary Table S1). Interestingly, the largest overall change in solvent accessibility is noted for βHis146(HC3): from 44% exposed in the R state to 0% exposed in the T state.

The physiologically relevant alkaline Bohr effect is a decrease in oxygen affinity as the arterial Hb at pH ∼7.4 encounters the pH of ∼7.2 in the peripheral tissue, allosterically promoting oxygen release in the transition from the R state to the T state. During this R-to-T-state transition, the binding of an estimated two protons per Hb tetramer occurs (Perutz *et al.*, 1980 ▸). Changes in His p*K*_a_ owing to the structural transition will either support proton uptake (negative Δp*K*_a_ on transitioning from T to R) or oppose proton uptake (positive Δp*K*_a_ on transitioning from T to R). As Hb returns to higher O_2_ concentrations, the increased oxygen tension promotes proton release and the subsequent increase in oxygen affinity known as the Haldane effect. During the normal delivery of oxygen, Hb retains two ligands on average, adopting a mixed population of the T state with two ligands on the same dimer and the R state with two ligands on opposing dimers (Ackers, 1980 ▸).

Allosteric effectors of Hb, such as 2,3-BPG, enhance the stability of the T-state tetramer, promoting ligand release. In the HuDeoxyHb T state, the βHis146(HC3) C-terminus forms a salt bridge to βAsp94(FG1) during the R-to-T transition. NMR studies determined that βHis146(HC3) has a negative Δp*K*_a_ from T to R (−1.51 by NMR, −1.1 by H/D exchange), contributing to the alkaline Bohr effect (Fang *et al.*, 1999 ▸; Sun *et al.*, 1997 ▸; Ohe & Kajita, 1980 ▸). This observation is partly supported by the neutron structures. In the T-state model, HuDeoxyHb β_1_His146(HC3) was determined to be doubly protonated and β_2_His146(HC3) was determined to be singly protonated. EqCNmetHb (R state) has βHis146(HC3) solvent-exposed with a high freedom of motion and it is likely to be singly protonated (His^0^). While both HuDeoxyHb βHis146(HC3) residues are proposed to form ‘intramolecular salt bridges’ with βAsp94(FG1), encountering nearly identical hydrogen-bonding networks, no nuclear density was observed at the N^δ1^ position of β_2_His146(HC3). A subtle difference between the two HuDeoxyHb βHis146(HC3) residues is the N⋯O hydrogen-bond distance to βAsp94(FG1), which is 2.8 Å in the doubly protonated β_1_ and 3.0 Å in the singly protonated β_2_. The shorter distance is likely to be promoted by the added electrostatic interaction of His^+^ with Asp in β_1_; however, both remain at distances normally assigned as hydrogen bonds (2.8–3.2 Å) and are not in the electrostatic range (2.4–2.6 Å). The neighboring His located at the end of the H helix, βHis143(H21), has been reported to have a p*K*_a_ that becomes more acidic from T to R (+0.87 by NMR, +0.5 by H/D-exchange studies; Fang *et al.*, 1999 ▸; Ohe & Kajita, 1980 ▸) and is predicted to be singly protonated in both the T and the R states. In the R state, βHis143(H21) appears to be singly protonated. In the T state, the dimers adopt states that are exactly the opposite of their βHis146(HC3) counterparts, with β_1_His143(H21) singly protonated and β_2_His143(H21) doubly protonated. All structural evidence suggests that these two neighboring residues work in concert to contribute to the overall Bohr effect.

The remaining potential Bohr groups are mostly surface residues and the protonation states of surface residues are heavily influenced by the pH of the solvent. Both states crystallized in monoclinic space groups, HuDeoxyHb in *P*2_1_ (with a tetramer in the asymmetric unit) and EqCNmetHb in *C*2 (with a dimer in the asymmetric unit) and, while related, the two exhibit different crystal contacts, potentially affecting their protonation states (Kovalevsky, Chatake *et al.*, 2010 ▸). Adding to the complexity for comparison is the fact that the HuDeoxyHb crystal was grown at pH 6.3 (pD 6.7), increasing the ratio of protonated to deprotonated (∼1:1) His residues on the surface (Chatake *et al.*, 2007 ▸). To contribute to the alkaline Bohr effect, a histidine residue must be singly protonated in the R state (His^0^) and become doubly protonated in the T state (His^+^). The NMR studies identified three residues that support the alkaline Bohr effect: αHis89(FG1) (Δp*K*_a_ = −0.55 by NMR and −1.6 by H/D exchange), βHis97(FG4 switch) (Δp*K*_a_ = −0.26 by NMR and no change by H/D exchange) and βHis146(HC3) (Δp*K*_a_ = −1.51 by NMR and −1.1 by H/D exchange) (Fig. 2 ▸; Sun *et al.*, 1997 ▸; Fang *et al.*, 1999 ▸; Ohe & Kajita, 1980 ▸). In comparing the two neutron structures, three residues in the α chain and one in the β-chain have protonation changes that support the alkaline Bohr effect: αHis72(EF1), αHis103(G10) and αHis112(G19), and βHis97(FG4 switch), respectively. For the first α residue, the HuDeoxyHb αHis72(EF1) T state is doubly protonated, while αHis72(EF1) is only protonated at the N^∊2^ position in the EqCNmetHb R state. In both states, αHis72(EF1) is exposed to solvent and does not exhibit any hydrogen bonds; therefore, the differences seen can be attributed to differences in the pH of the crystallization buffers.

**Figure 3:**
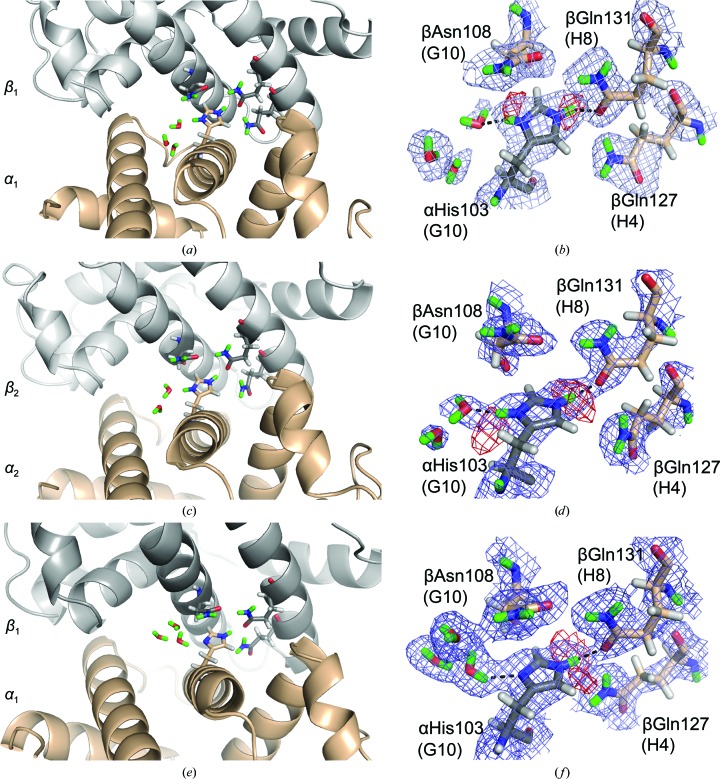
αHis103(G10) in the α_1_:β_1_ interface supports the alkaline Bohr effect: T-state HuDeoxyHb α_1_:β_1_ interface (*a*, *b*), T-state HuDeoxyHb α_2_:β_2_ interface (*c*, *d*) and R-state EqCNmetHb α_1_:β_1_ interface (*e*, *f*). The α:β dimer interface indicates the position of αHis103, with the α subunits in wheat and the β subunits in gray (*a*, *c*, *e*). The electron and neutron density maps (*b*, *d*, *f*) display the H^+^/D^+^ states of αHis103(G10), which is doubly H^+^/D^+^-occupied in the T state with disrupted hydrogen-bond networks to βAsn108(G10), βGln127(H4) and βGln131(H8) (*b*, *d*) and singly H^+^/D^+^-occupied in the R state (*f*) with a rigid hydrogen-bond network. Here and in all figures, the nuclear density 2|*F*_o_| − |*F*_c_| maps are in blue, contoured at 1.0σ. OMIT nuclear density |*F*_o_| − |*F*_c_| maps are in red, contoured at 2.0σ. The exchanged D atoms are displayed in green, H atoms in white, N atoms in blue and O atoms in red; the black dashed lines indicate hydrogen bonds.

The second α residue, αHis103(G10), is located within the α_1_:β_1_ interface (solvent accessibility = 0) near the hinge region on the F helix of hemoglobin and is the most intriguing of those that support the alkaline Bohr effect. In the NMR studies, the p*K*_a_ shifts were not determined for αHis103(G10) owing to the incorporation of the α_1_:β_1_ interface into the core (Sun *et al.*, 1997 ▸; Fang *et al.*, 1999 ▸). In the H/D-exchange experiment, the p*K*_a_ of αHis103(G10) was determined to be 7.1 and was unaffected by the change of state, predicting partial occupancy in the T state (HA:A ratio of 1:0.7). Calculations estimate a strongly acidic p*K*_a_ (3.2 in the T state and 3.9 in the R state) precluding any involvement in the Bohr effect (Zheng *et al.*, 2013 ▸). Visually, both α_1_His103(G10) and α_2_His103(G10) in the HuDeoxyHb T state were determined to be doubly protonated with full occupancy (Figs. 3 ▸ *a*–3 ▸ *d*). The EqCNmetHb R-state α_1_His103(G10) is singly protonated (Figs. 3 ▸ *e* and 3 ▸ *f*). In the EqCNmetHb R state, the N^δ1^ atom of αHis103(G10) acts as a hydrogen-bond acceptor to a water which is bridged by another water to the side-chain N atom of βAsn108(G10). In the HuDeoxyHb T state, the N^δ1^ atom of αHis103(G10) acts as a hydrogen-bond donor to the nearest water and the coupling to βAsn108(G10) is lost. The hydrogen-bond network coupling αHis103(G10) to βAsn108(G10) bridges the G and H helices, involving βGln127(H4) and βGln131(H8). All four of these hydrogen-bond-linked residues are highly conserved and have known clinical variants. The βGln127(H4)→Arg (Hb Dieppe) and βGln131(H8)→Arg variants are reported to be highly unstable since a positive charge is being buried in the α_1_:β_1_ dimer interface (Girodon *et al.*, 1992 ▸; Beneitez Pastor *et al.*, 2007 ▸). More intriguing are the αHis103(103)→Arg (Hb Contaldo) and βAsn108(G10)→Ile (Hb Schlierbach) variants, which form unstable and low O_2_ affinity Hbs, respectively (Sciarratta *et al.*, 1984 ▸; Frischknecht *et al.*, 1999 ▸). Both mutations would disrupt the R-state hydrogen-bond network and the destabilization promoting the adoption of the low-affinity T state.

**Figure 4:**
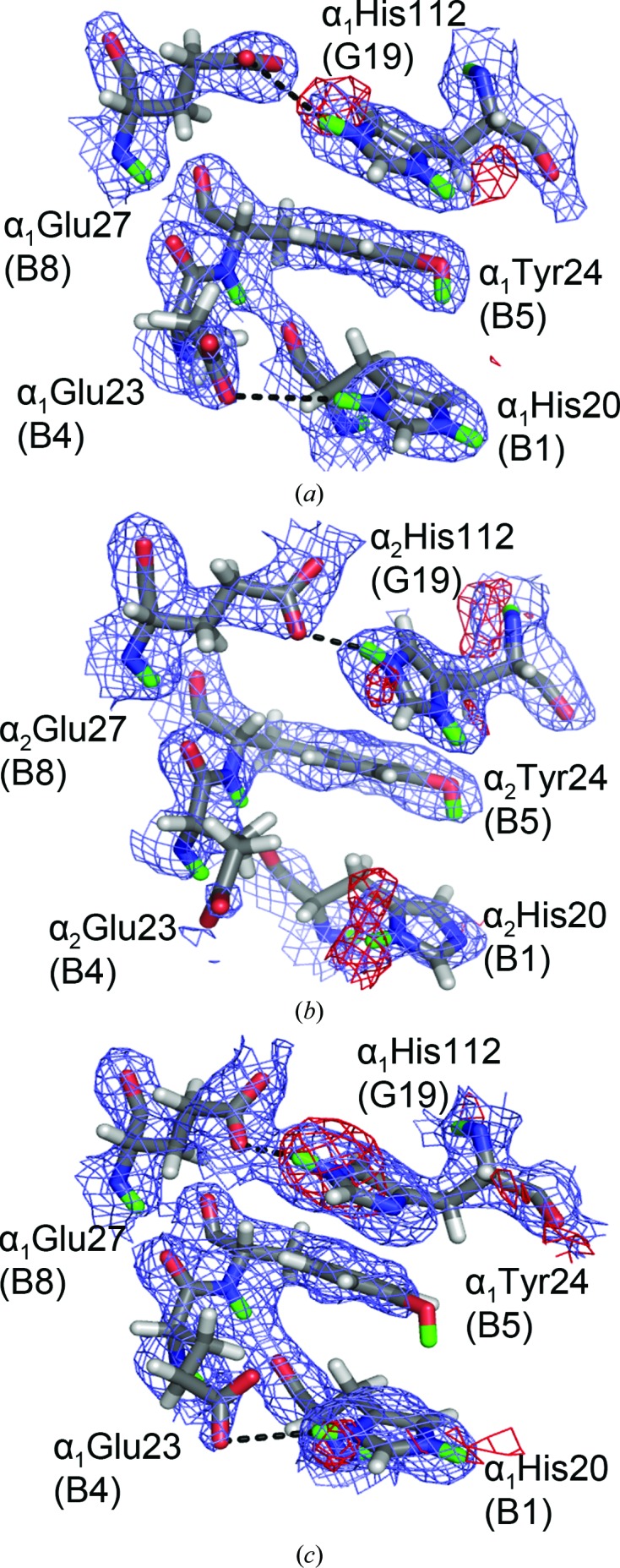
Alternate protonation states of αHis20(B1) and αHis112(G19). Located on the surface near the α_1_:β_1_ interface, αHis20(B1) is doubly protonated in T-state HuDeoxyHb α_1_ (*a*), singly protonated in T-state HuDeoxyHb α_2_ (*b*) and doubly protonated in R-state EqCNmetHb α (*c*), while αHis112(G19) is doubly protonated in T-state HuDeoxyHb α_1_ and α_2_ (*a*, *b*) and singly protonated in R-state EqCNmetHb α (*c*).

**Figure 5:**
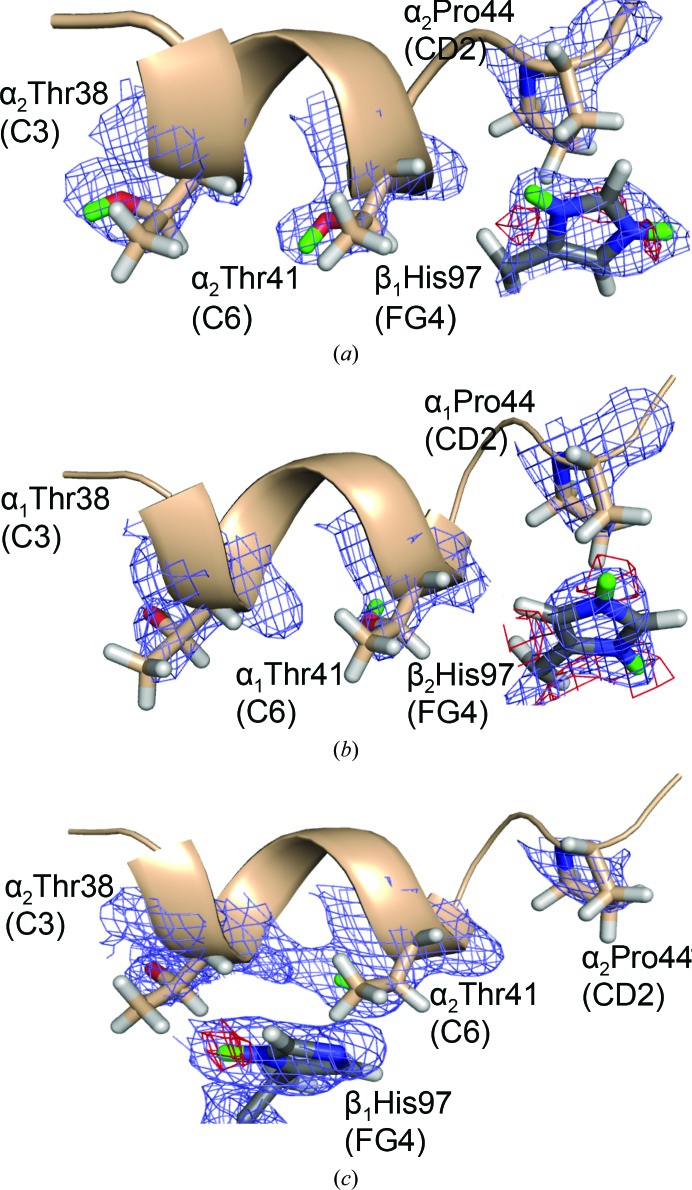
βHis97(FG4) supports the alkaline Bohr effect: βHis97(FG4) is located in the switch region of the α_1_:β_2_ interface. In T-state HuDeoxyHb (*a*, *b*), βHis97(FG4) is tightly positioned between αThr41(C6) and αPro44(CD2) and is doubly protonated. In R-state EqCNmetHb (*c*), βHis97(FG4) is loosely positioned between αThr38(C3) and αThr41(C6) and is singly protonated.

For the third α residue, HuDeoxyHb T-state α_1_His112(G19) and α_2_His112(G19) are both doubly protonated, while in the EqCNmetHb R state the N^δ1^ D atom is absent (Fig. 4 ▸). In both states, αHis112(G19) is hydrogen-bonded to αGlu27(B8); the N⋯O distances are 2.8 Å (EqCNmetHb) and 3.4 Å (HuDeoxyHb). In the case of HuDeoxyHb αHis112(G19), the protonation state can be attributed to the hydrogen-bond interactions with αGlu27(B8) and a weak hydrogen bond to αTyr24(B5). Interestingly, this interaction seems to affect the capability to observe the D atom on αTyr24(B5). With the loss of the hydrogen bond between αTyr24(B5) and αHis112(G19) in the R state, an increase in the freedom of motion of the tyrosyl phenol D atom of αTyr24(B5) is observed, as indicated by the refined negative occupancy. The only potential Bohr group in the β chain, βHis97(FG4 switch), appears to be doubly protonated in the HuDeoxyHb T state, and is located in close proximity to αThr41(C6) and αPro44(CD2) in the switch region of the hemoglobin tetramer (Figs. 5 ▸ *a* and 5 ▸ *b*). In the EqCNmetHb R state, βHis97(FG4) is singly protonated and is further away from the α_1_/β_2_ interface, located between αThr38(C3) and αThr41(C6) but exposed to solvent (Fig. 5 ▸ *c*).

**Figure 6:**
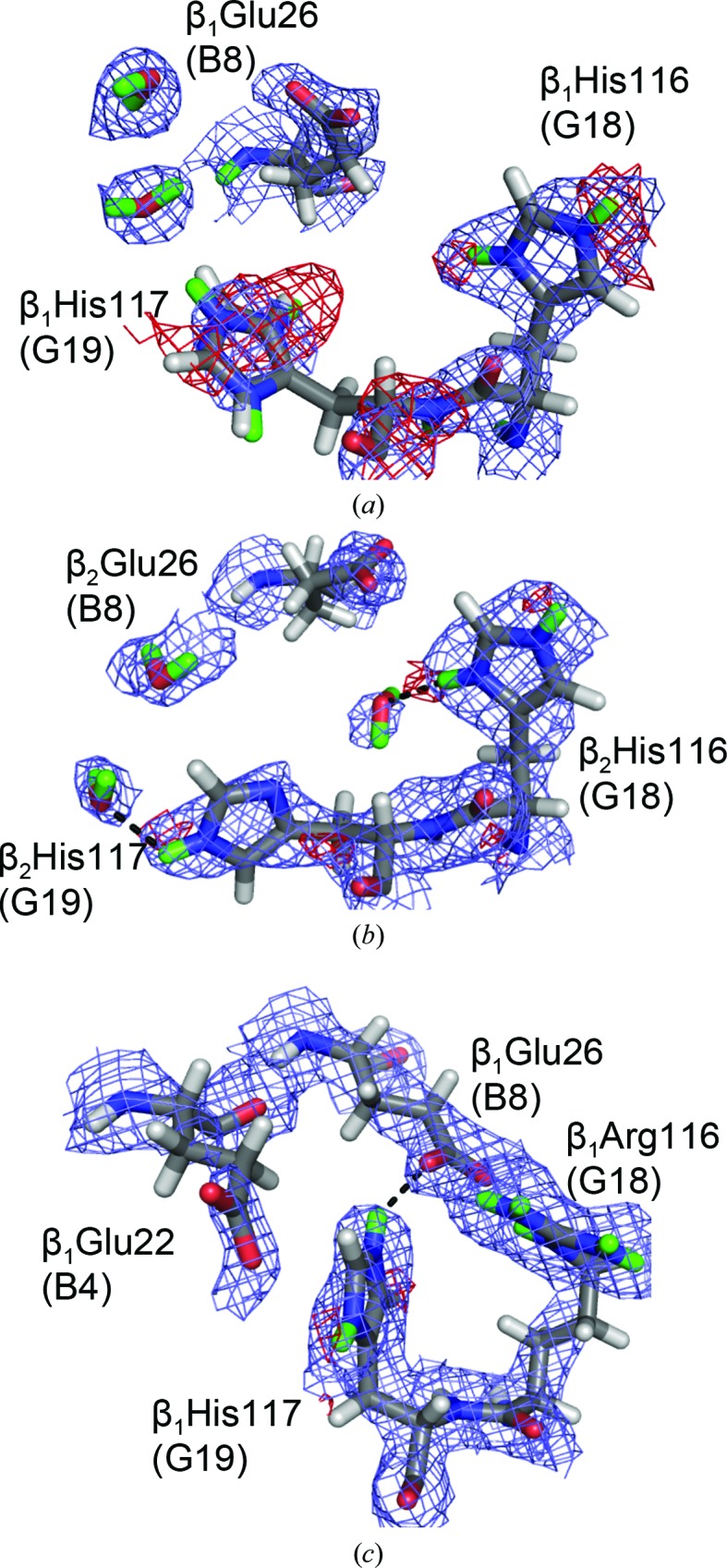
βHis117(G19) counters the alkaline Bohr effect. Solvent-exposed and located near the end of the G helix, βHis117(G19) appears to be singly protonated with dual conformation in T-state HuDeoxyHb β_1_ (*a*), singly protonated in the T-state HuDeoxyHb β_2_ chain (*b*) and doubly protonated in R-state EqCNmetHb β (*c*). βHis116(G18) is doubly protonated in the T state (*a*, *b*) but is not conserved (*c*).

In addition to βHis143(H21) as discussed above, the NMR studies identified three additional residues that could potentially counter the alkaline Bohr effect: αHis20(B1) (Δp*K*_a_ = +0.06 by NMR and −0.6 by H/D exchange), αHis112(G19) (Δp*K*_a_ = +0.04 by NMR and no change by H/D exchange) and βHis77(EF1) (Δp*K*_a_ = +0.6 by NMR and no change by H/D exchange) (Fig. 2 ▸; Sun *et al.*, 1997 ▸; Fang *et al.*, 1999 ▸; Ohe & Kajita, 1980 ▸). When comparing the two neutron structures, two different His residues were identified, αHis20(B1) and βHis117(G19), which were determined to be doubly protonated in the R state and singly protonated in the T state. The solvent-exposed αHis20(B1) is weakly hydrogen-bonded to αGlu23(B4) (Fig. 4 ▸). In EqCNmetHb αHis20(B1) is protonated at both the N^δ1^ and N^∊2^ positions, in which N^δ1^ acts as a weak hydrogen-bond donor to αGlu23(B4) (N⋯O distance of 3.5 Å). The HuDeoxyHb αHis20(B1) is doubly protonated in α_1_ (N⋯O distance of 4.5 Å) but not in α_2_ (6.2 Å N⋯O distance), losing hydrogen bonds to αGlu23(B4) in both the α_1_ and α_2_ subunits. In EqCNmetHb, βHis117(G19) is clearly protonated at both the N^δ1^ and N^∊2^ positions, as shown by the 2*F*_o_ − *F*_c_ nuclear density map (Fig. 6 ▸). In HuDeoxyHb, both the β_1_His117(G19) and β_2_His117(G19) were determined to not be protonated and the β_1_His117(G19) protonation state was justified by the side chain being disordered. In EqCNmetHb βHis117(G19) N^∊2^ donates a hydrogen bond to βGlu26(B8) (N⋯O distance of 3.0 Å), while in the HuDeoxyHb neutron structure β_2_His117(G19) is rotated 180° and βGlu26(B8) is 7.0 Å away from the N^δ1^ position of β_2_His117(G19). Also, in EqCNmetHb βHis117(G19) N^δ1^ makes a weak electrostatic interaction with βGlu22(B4) (N⋯O distance of 3.4 Å) and in HuDeoxyHb βGlu22(B4) is more than 8.0 Å away (Fig. 6 ▸). It is important to note that βHis116(G18) in HuDeoxyHb is not conserved in equine Hb [βArg116(G18)]; both side chains contain a positive charge but allow distinctive interactions to occur between the two states.

In summary, for most of the His residues in the R state His^0^ is prevalent as expected, whereas the T state adopts a mixture of single and double protonation states, as was observed for βHis143(H21) and βHis146(HC3), including residues αHis50(CD8), αHis58(E7 distal), αHis89(FG1), βHis2(NA2) and βHis63(E7 distal). The His residues that have no changes in apparent protonation state are αHis45(CD3), αHis87(F8 proximal), αHis122(H5), βHis77(EF1) and βHis92(F8 proximal). Here, we report several His residues that do contribute to the alkaline Bohr effect, including αHis103(G10), αHis112(G19) and βHis146(HC3), and two His residues that counteract the Bohr effect, αHis20(B1) and βHis117(G19). It is important to note that no other types of residues exhibited changes in protonation state that are related to the Bohr effect. Directly determining the atomic position of D atoms that are bonded to His residues contributes to the understanding of how H^+^ can act as a heterotopic modulator. Interestingly, this is the first time that αHis103(G10), an α_1_:β_1_ interface contact, has been shown to have a definitive structural linkage to the Bohr effect where NMR, H/D exchange and calculations anticipate no differences.

### 3.3. Comparison of R and T states and the distal histidine   

**Figure 7:**
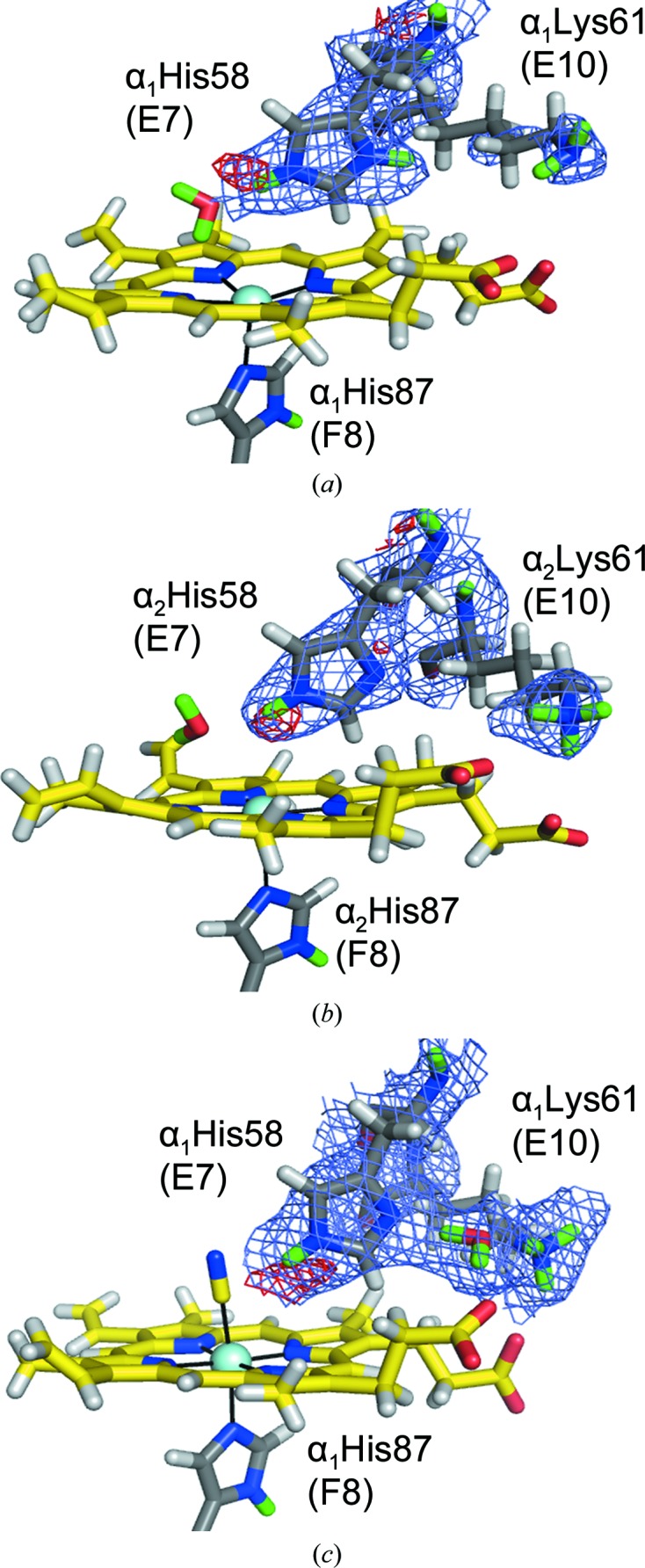
The distal histidines, α_1_His58(E7) and β_1_His63(E7): alternate protonation states. In the α_1_β_1_ dimer of T-state HuDeoxyHb both distal histidines are doubly protonated (*a*) (α_1_ is shown, but β_1_ is similar), while in the α_2_β_2_ dimer of T-state HuDeoxyHb (*b*) (α_2_ is shown, but β_2_ is similar) both distal histidines are singly protonated. With the presence of ligand in R-­state EqCNmetHb (*c*) (α is shown, but β is similar), the coordinated water molecule is displaced, the singly protonated distal His is hydrogen-bonded to the ligand and Lys(E10) becomes coupled to the heme proprionate group and to His(E7) through a bound water.

Another unique difference between the R and T states (EqCNmetHb and HuDeoxyHb) is the change in distal His(E7) protonation states. The HuDeoxyHb neutron structure indicates that the protonation state of the distal His varies between the two dimers: α_1_β_1_ His(E7) residues are doubly protonated while α_2_β_2_ His(E7) residues are singly protonated. All distal His(E7) residues in HuDeoxyHb have a D_2_O molecule accepting a D atom connected to the N^∊2^ atom by a hydrogen bond and forming O—H⋯π contacts with the hemes. Curiously, in the α_1_β_1_ dimer the distal His residues [α_1_His58(E7) and β_1_His63(E7)] are doubly protonated while the neighboring Lys residues [α_1_Lys61(E10) and β_1_Lys66(E10)] were determined to be deprotonated (ND_2_) (Fig. 7 ▸ *a*). The opposite was observed in the α_2_β_2_ dimer: α_2_His58(E7) and β_2_His63(E7) are singly protonated while the neighboring Lys residues [α_2_Lys61(E10) and β_2_Lys66(E10)] were determined to be protonated (N^δ1^) (Fig. 7 ▸ *b*).

In the R-state neutron structure, both of the distal His residues [αHis58(E7) and βHis63(E7)] are singly protonated (Fig. 7 ▸ *c*). In the presence of ligand, greater order in the region of the distal histidine appears, with a water molecule that is not present in the T state positioned between the distal His(E7) and Lys(E10). The deprotonated N^δ1^ atom of the distal His(E7) accepts a hydrogen bond from this bridging water, while Lys(E10) donates a hydrogen bond (Fig. 7 ▸ *c*). The ordering of Lys(E10) stabilizes its coupling to the heme propionate group, an interaction that is substantially weaker in the T state. The protonation states of the distal histidine residues do not support their contribution to the Bohr effect. However, this is the first time that any evidence of a possible proton transfer linked to the distal histidine that occurs between the two states of hemoglobin has been reported.

### 3.4. Comparison of R and T states and backbone amide H/D exchange   

**Figure 8:**
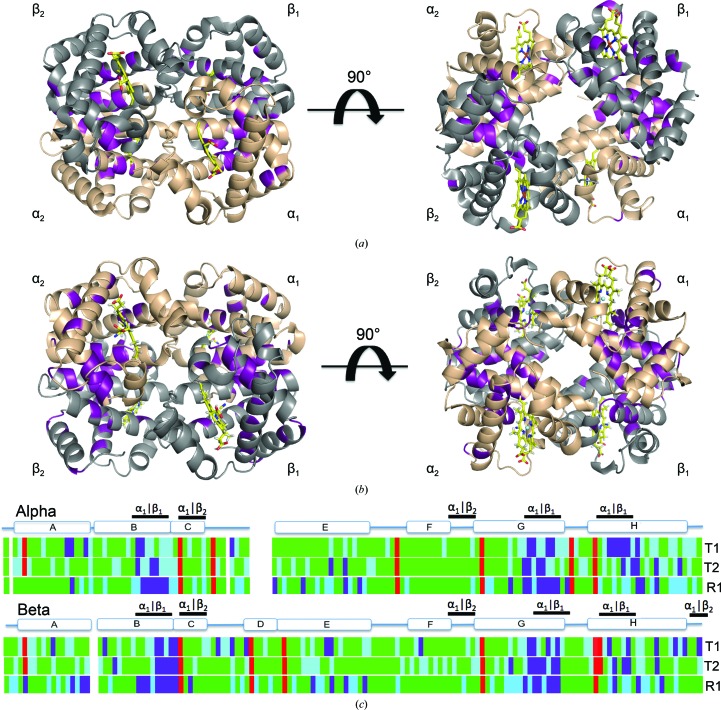
Amide H/D exchange indicates that T-state HuDeoxyHb has greater flexibility, while R-state EqCNmetHb displays less exchange in the αβ dimer interface. Superimposed on the ribbon figures of T-state HuDeoxyHb (*a*) and R-state EqCNmetHb (*b*) are the positions of non-exchanged backbone amide H atoms (magenta) indicating regions of minimal flexibility. The T state has regions of non-exchange in the α_1_β_2_ tetramer interface, while the R state has a more extensive region of non-exchange in the α_1_β_1_ dimer interface. (*c*) shows the H/D exchange related to the primary sequence, with T1 and T2 indicating the two dimers of the T-state HuDeoxyHb model and R indicating the R-state EqCNmetHb model, and is color-coded as follows: non-exchanged in magenta (occupancy range −0.56 to 0.0), partially exchanged in cyan (occupancy range 0.0–0.6) and fully exchanged in green (occupancy range 0.6–1.0); prolines indicated in red lack backbone amide protons. The secondary structure and interface contacts are aligned with the sequence.

The partial double-bond character of the backbone amide bond allows H/D exchange (with the exception of proline) through acidic (protonation of N), basic (deprotonation of N) and imidic acid (protonation of O) mechanisms. This provides information pertaining to regions of flexibility that allow exchange and to regions of stability where exchange is prohibited (Engen, 2009 ▸). The two crystals used in the neutron diffraction experiments were mounted in quartz capillaries and exposed to D_2_O by vapor diffusion for a minimum of one month before data collection. The HuDeoxyHb mother liquor had a pH of 6.3 (pD 6.7), while the EqCNmetHb mother liquor had a pH of 7.2 (pD 7.6). The rate of exchange is affected by the pH/pD of the solution, with rates decreasing at lower pH (Connelly *et al.*, 1993 ▸). The rates of exchange for pD 6.7 and pD 7.6 are not significantly different. The two crystals were crystallized and exchanged with deuterium using different methods. HuDeoxyHb underwent buffer exchange with D_2_O for several hours (∼1 × 10^−4^ s^−1^) before the deoxygenation step, followed by crystallization, which required several days, while EqCNmetHb was exchanged with D_2_O after crystallization (Kovalevsky, Chatake *et al.*, 2010 ▸; Kovalevsky, Fisher *et al.*, 2010 ▸). This indicates that the highly reactive backbone amide protons (exchange rates lower than 1 × 10^−4^ s^−1^) cannot be the subject of this analysis. Instead, this comparison focuses on the protected backbone amide protons with partial exchange (rates of ∼10^−5^–10^−6^ s^−1^) and with no exchange (rates of >3 × 10^−6^ s^−1^) (Englander, 1975 ▸). HuDeoxyHb has 56.8% of the amide protons fully exchanged (occupancy 0.6–1.0), 26.7% of the amide protons partially exchanged (occupancy 0.0–0.6) and 11.8% with no exchange (occupancy −0.56 to 0.0) (Fig. 8 ▸ *a*). EqCNmetHb has 56.1% fully exchanged, 22.6% partially exchanged and 17.1% with no exchange (Fig. 8 ▸ *b*).

Overall, the HuDeoxyHb T state has 0.7 and 4.1% more fully and partially exchanged main-chain amides, respectively, *versus* the EqCNmetHb R state. This may be due in part to the differences in D_2_O exposure prior to crystallization that would primarily affect the rapidly exchanging protons. While no controlled studies on the comparison of vapor *versus* liquid exchange exist, the extensive studies of xylose isomerase (XI) offer some insight. For the nine neutron structures of XI (PDB entries 3cwh, 4dvo, 3kcj, 3qza, 2gve, 4qdw, 4qdp, 3kcl and 3kco), the percentage non-exchanged varied from 9 to 36%, where two were liquid-exchanged (PDB entries 3chw and 2gve) and the rest were vapor-exchanged. As might be expected if differences exist, PDB entry 3chw does have the most exchange. However, PDB entry 2gve was subjected to several months of liquid exposure prior to microgravity crystallization as part of a NASA Shuttle mission (Katz *et al.*, 2006 ▸). At 30.8%, PDB entry 2gve is in the group that are least exchanged. These XI results suggest that liquid and vapor exchange are comparable techniques, at least for comparing the non-exchanged regions. For the hemoglobin neutron structures, the T-state and R-state amide H/D exchanges are depicted relative to the sequence (Fig. 8 ▸ *c*). In the HuDeoxyHb T state, the primary areas of stability are localized in the α_1_:β_2_ and α_2_:β_1_ interfaces (Figs. 8 ▸ *a* and 8 ▸ *c*). The presence of ligand in the EqCNmetHb R state shifts stability to the cores of the individual monomers and extends the stability of the α_1_:β_1_ interface (Figs. 8 ▸ *b* and 8 ▸ *c*). These results are in agreement with Acker’s free-energy penalty model of ligand binding (Ackers, 1980 ▸). Hemoglobin is a passive transporter where strain is not lost or gained but shifted from one region to another when switching between states. As ligands are lost, the interfaces forming the tetramer in the T state are stabilized. As ligands bind, the R-state monomer interactions are stabilized at the expense of the dimer–dimer interface.

## Supplementary Material

PDB reference: neutron structure of equine cyanomethemoglobin, 5c6e

Supplementary Table S1.. DOI: 10.1107/S2059798316009049/mn5110sup1.pdf

## Figures and Tables

**Table 1:** Neutron crystallographic data-collection and refinement statistics

	Neutron	X-ray
Data collection		
No. of crystal settings	37	
Space group	*C*2	*C*2
Unit-cell parameters (Å, °)	*a* = 108.86, *b* = 63.16, *c* = 54.71, β = 110.75	*a* = 108.76, *b* = 63.04, *c* = 54.58, β = 110.73
Resolution	21.58–2.00 (2.11–2.00)	30.20–1.70 (1.79–1.70)
No. of reflections	51389	137361
Unique reflections	18780	35839
Multiplicity	2.7 (1.9)	3.8 (3.8)
Completeness (%)	80.2 (67.9)	94.6 (91.1)
〈*I*/σ(*I*)〉	3.5 (1.4)	12.9 (3.9)
*R*_merge_ †	0.242 (0.356)	0.064 (0.287)
*R*_p.i.m._ ‡	0.163 (0.283)	0.039 (0.168)
Joint XN refinement		
Resolution (neutron) (Å)	21.58–2.00	
Resolution (X-ray) (Å)	30.20–1.70	
Data-rejection criteria	No observation and |*F*| = 0	
Sigma cutoff	2.5	
*R*_work_ §/R_free_ ¶ (neutron)	0.301/0.334	
*R*_work_ §/R_free_ ¶ (X-ray)	0.210/0.2329	
No. of protein atoms	4403	
No. of D_2_O molecules	123	
R.m.s.d., bond lengths (Å)	0.008	
R.m.s.d., bond angles (°)	1.09	
Average *B* factors (Å^2^)		
Main chain	33.5	
Side chain	35.9	
Solvent	49.8	
Heme groups	32.9	

† *R*_merge_ = 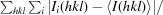 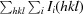.

‡ *R*_p.i.m._ = 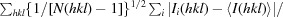 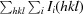.

§ *R*_work_ = 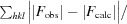 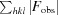.

¶ *R*_free_ is calculated in the same way as *R*_work_ for data omitted from refinement (5% of reflections for all data sets).
