# Supplementary material for: Visualizing the Bohr effect in hemoglobin: neutron structure of equine cyanomethemoglobin in the R state and comparison with human deoxyhemoglobin in the T state
Source: Acta Crystallogr D Struct Biol. 2016 Jun 28;72(Pt 7):892–903. doi: 10.1107/S2059798316009049 (PMC4932920; doi:10.1107/S2059798316009049)
Supplement: Supplementary file 1 [file d-72-00892-sup1.pdf]

STRUCTURAL
BIOLOGY

Volume 72 (2016)

Supporting information for article:

Visualizing the Bohr effect in hemoglobin: neutron structure of equine cyanomet-hemoglobin in the R-state and comparison with human deoxy-hemoglobin in the T-state

Steven Dajnowicz, Sean Seaver, B. Leif Hanson, S. Zoë Fisher, Paul Langan, Andrey Y. Kovalevsky and Timothy C. Mueser

Table S1 Solvent Accessibility

The percent solvent accessibility for each of the His residues in the R-and-T-state: N/A = not applicable.

His #	HbCNmet α	Deoxy-Hb α_1	Deoxy-Hb α_2
20	82	69	75
45	7	11	25
50	93	81	46
58 Distal	0	0	0
72	79	72	56
87 Proximal	0	0	0
89	52	52	43
103	0	0	0
112	38	18	25
122	0	0	0
His#	β	β_1	β_2
2	N/A	N/A	N/A
63 Distal	0	0	0
69	N/A	N/A	N/A
76	N/A	N/A	N/A
77	80	36	91
92 Proximal	0	0	0
97	58	48	39
116	N/A	N/A	N/A
117	0	11	5
143	45	44	66
146	44	0	2
